# Association Between Metabolic Syndrome and Choledocholithiasis: An Observational, Analytical, Retrospective Cross-Sectional Study Conducted at a Second-Level Hospital in Ciudad Juárez From January 2024 to February 2025

**DOI:** 10.7759/cureus.86637

**Published:** 2025-06-24

**Authors:** Yareli Lizbeth Rojas Salazar, Emiliano Gomez Montanez, Jorge Gustavo Rojas Salazar

**Affiliations:** 1 Cancer Functional Genomics Laboratory, National Institute of Genomic Medicine, Mexico City, MEX; 2 General Surgery, Autonomous University of Ciudad Juárez, Ciudad Juárez, MEX

**Keywords:** body mass index, choledocholithiasis, metabolic syndrome, obesity, risk factors

## Abstract

Introduction

Metabolic syndrome (MS) is a disorder that groups conditions such as central obesity, hypertension, dyslipidemia, and hyperglycemia, and it is also associated with cardiovascular diseases and type 2 diabetes mellitus, in which an increase in biliary diseases such as choledocholithiasis, characterized by the presence of stones in the common bile duct, has been observed. This association may be explained by metabolic alterations that lead to increased cholesterol saturation in bile and impaired gallbladder motility due to insulin resistance, both of which contribute to the formation of stones. This study aims to investigate the relationship between MS and choledocholithiasis in patients treated at a second-level hospital in Ciudad Juárez between January 2024 and February 2025.

Materials and methods

Observational, analytical, retrospective, cross-sectional study with 59 patients over 17 years of age who were divided into two groups: with choledocholithiasis (29, 49%) and without choledocholithiasis (30, 51%). For each group, the ATP III diagnostic criteria for MS were evaluated. For statistical analysis, chi-square, Fisher’s exact test, and odds ratio were used.

Results

The presence of MS was significantly associated with choledocholithiasis (p=0.002; OR=5.83, 95% CI: 1.93-19.33); BMI over 30 was the only MS component with a statistically significant association in both patients with and without choledocholithiasis (p=0.0016 and p=0.008, respectively).

Conclusions

The presence of MS is a risk factor for the development of choledocholithiasis in the studied population. A high BMI is confirmed as a relevant risk factor. These findings provide useful local evidence for the development of preventive strategies and clinical management.

## Introduction

Metabolic syndrome (MS) is defined as a group of metabolic alterations that include central obesity, arterial hypertension, dyslipidemia, and hyperglycemia, which collectively increase the risk of developing cardiovascular diseases and type 2 diabetes mellitus. Its prevalence varies according to country, ethnicity, and the diagnostic criteria used [1]. The prevalence of MS is 25%, while in Latin America it is estimated to be between 30% and up to 40%, and it has been increasing in recent decades due to lifestyle and eating habit changes [2].

On the other hand, an increase in the prevalence of biliary diseases, such as cholelithiasis, cholecystitis, and choledocholithiasis, has also been observed. The latter is defined as the presence of gallstones in the common bile duct, which can lead to severe complications, including cholangitis, biliary pancreatitis, and biliary obstruction [3]. It has been suggested that MS may be related to the pathophysiology of choledocholithiasis, possibly due to factors such as insulin resistance, altered bile composition, and increased biliary cholesterol [4].

Some studies have analyzed the relationship between each of the components that make up MS and gallstone disease; however, there is not much evidence studying the association between MS as a complete clinical entity and the development of choledocholithiasis, so this remains a field with considerable opportunity for research, and within the available evidence, most focus on cholelithiasis in general, leaving aside knowledge about choledocholithiasis [5]. Some proposed mechanisms that may explain this association include alterations in bile composition, for example, increased cholesterol saturation, as well as impaired gallbladder motility linked to insulin resistance, both of which are commonly observed in individuals with MS [5]. Another factor to consider is that in local populations, such as those in Ciudad Juárez, there is insufficient evidence to document this association, thereby limiting the establishment of targeted prevention and management strategies.

Given the above, this study aims to explore the possible association between MS, a clinical entity, and the presence of choledocholithiasis in patients treated at a hospital in Ciudad Juárez, thereby providing local evidence to enhance current knowledge.

## Materials and methods

Study design

An observational, analytical, retrospective cross-sectional study was conducted. The study population consisted of patients over 17 years of age who were treated in a second-level hospital in Ciudad Juárez between January 2024 and February 2025. The sample size consisted of 59 patients, based on the total number of cases available in the hospital database; among these, 29 patients were diagnosed with choledocholithiasis, while 30 patients did not have this diagnosis. The sample was selected through non-probabilistic convenience sampling, including all records that met the inclusion and exclusion criteria established for the study; and given the sample size and the sampling approach, it is acknowledged that the findings may be subject to certain limitations, particularly in terms of representativeness and the possibility of selection-related distortions.

Inclusion and exclusion criteria

This study included patients of both sexes over 17 years of age with a confirmed diagnosis of choledocholithiasis, established through imaging studies such as ultrasound, cholangiography, or computed tomography, or surgical findings; additionally, only those records that contained sufficient information to evaluate the components of MS, specifically blood pressure measurements, lipid profile, blood glucose levels, and anthropometric data, were considered eligible.

Patients were excluded if they had a history of previous biliary tract surgery or any known biliary tract disease prior to the current episode. Furthermore, records that were incomplete and did not provide enough data to determine the presence or absence of MS were also excluded from the study.

Instruments used

For data collection, an institutional database provided by the second-level hospital in Ciudad Juárez was utilized, comprising clinical records of patients over 17 years of age treated between January 2024 and February 2025. The records included information from 59 patients regarding the diagnosis of choledocholithiasis, as well as clinical parameters such as blood pressure, fasting glucose, triglycerides, high-density lipoprotein (HDL) cholesterol, BMI, and sociodemographic data (age and sex).

Statistical analysis

Data were analyzed using the statistical program R Studio version 2024.12.1 (Posit PBC, Boston, MA, USA). The variables considered were the presence of MS according to the ATP III criteria, the presence of choledocholithiasis, and sociodemographic variables, including age and sex.

A descriptive analysis of the qualitative variables was conducted using percentages and of the quantitative variables through means and standard deviations. For inferential analysis, the association between MS and choledocholithiasis was established using the chi-square test (χ²). For the association between MS and the variables age, sex, and BMI, Fisher's test was used due to expected frequencies less than five, considering a p-value <0.05 as statistically significant, in addition to the calculation of the odds ratio (OR) with a 95% confidence interval (CI).

Ethical considerations

This study was approved by the Coordination of the Medical Surgeon program of the Autonomous University of Ciudad Juárez and by the Teaching Department of the second-level hospital in Ciudad Juárez. The information used was obtained from anonymous institutional databases, thereby ensuring patient confidentiality in accordance with the ethical principles outlined in the Declaration of Helsinki. No intervention was carried out on the patients, since it was a retrospective analysis of clinical records.

## Results

Of the 59 patients evaluated, 43 (73%) were female and 16 (27%) were male. This predominance of female patients reflects the known higher prevalence of choledocholithiasis in women; however, it should be taken into account when interpreting the generalizability of the results. A total of 23 (39%) patients were in the 17- to 40-year age group, 28 (47%) were between 40 and 60 years old, and nine (15%) were over 60 years old. Twenty-nine (49%) patients were diagnosed with choledocholithiasis, while 30 (51%) were not diagnosed with choledocholithiasis. Regarding BMI, 13 (22%) patients were in the range of 18 to 25 kg/m², 25 (42%) were in the range of 25 to 30 kg/m², 12 (20%) were in the range of 30 to 35 kg/m², and nine (15%) were in the range of 35 to 40 kg/m². Given the role of obesity and altered lipid metabolism in MS, the BMI distribution is a relevant factor that may help explain its association with choledocholithiasis, especially in the absence of waist circumference measurements. Table 1 shows the mean, standard deviation, median, minimum, and maximum values for age, days of hospital stay, BMI, glucose, triglyceride levels, and HDL cholesterol in all evaluated patients; and although waist circumference is a standard criterion for MS diagnosis, BMI was used in this study as a substitution due to the unavailability of complete data; this substitution has been considered acceptable in similar studies, especially in individuals with a BMI higher than 30 kg/m².

**Table 1:** Descriptive analysis of age, days of hospital stay, BMI, glucose, triglycerides, and HDL cholesterol in all patients BMI: body mass index, HDL: high-density lipoprotein

	Mean	Standard deviation	Median	Minimum	Maximum
Age	44.96	14.90	46	17	85
Days of hospital stay	5.01	3.48	4	1	18
BMI	29.38	5.35	29	20	40
Glucose	104.96	35.54	95	61	242
Triglycerides	162	57.18	148	80	325
HDL cholesterol	50.08	14.52	50	20	80

Table 2 presents the mean age, hospital stay duration, BMI, glucose, triglyceride levels, and HDL cholesterol levels in all patients, categorized by the presence or absence of MS according to the ATP III criteria. Thirty-one patients did not meet the requirements for a diagnosis of MS, with a mean age of 45.2 years, a mean hospital stay of 4.48 days, a mean BMI of 25.9, a mean blood glucose level of 86.3 mg/dL, a mean triglyceride level of 120 mg/dL, and a mean HDL cholesterol level of 61.5 mg/dL. On the other hand, 28 patients did meet sufficient criteria for the diagnosis of MS, with a mean age of 44.7 years, a mean hospital stay of 5.61 days, a mean BMI of 33.3, a mean blood glucose of 126, a mean triglyceride level of 209, and a mean HDL cholesterol level of 37.4.

**Table 2:** Mean values of age, days of hospital stay, BMI, blood glucose, triglycerides, and HDL cholesterol in all patients separated by the presence or absence of MS MS: metabolic syndrome, BMI: body mass index, HDL: high-density lipoprotein

MS	Mean age	Mean length of stay (days)	Mean BMI	Mean blood glucose	Mean triglycerides	Mean HDL cholesterol
No	45.22	4.48	25.87	86.38	119.6	61.59
Yes	44.67	5.60	33.28	125.64	208.8	37.36

Table 3 shows the 2 x 2 contingency table to determine the statistically significant association between the presence of MS and choledocholithiasis. The chi-square test was used due to expected values greater than 5 in the contingency table, as well as the dichotomous nature, obtaining a chi-square value of 8.9522 with a p-value of 0.002, consistent with a statistically significant association, and an OR of 5.83 (95% CI: 1.93-19.33).

**Table 3:** 2 x 2 contingency table showing presence and absence of MS and choledocholithiasis MS: metabolic syndrome

	Presence of choledocholithiasis	Absence of choledocholithiasis	Total
Presence of MS	20	9	29
Absence of MS	8	22	30
Total	28	31	59

Table 4 presents the descriptive analysis of age, days of hospital stay, BMI, glucose, triglycerides, and HDL cholesterol in patients diagnosed with choledocholithiasis. No difference is observed between the minimum and maximum values, and thus the range, compared to Table 1, which includes all patients. Table 5 shows the mean age, days of hospital stay, BMI, glucose, triglyceride levels, and HDL cholesterol divided by the presence or absence of MS in patients with a diagnosis of choledocholithiasis, where no difference greater than 1 standard deviation was observed in any of the variables compared to Table 2 with all patients.

**Table 4:** Descriptive analysis of age, days of hospital stay, BMI, glucose, triglycerides, and HDL cholesterol in patients with choledocholithiasis BMI: body mass index, HDL: high-density lipoprotein

	Mean	Standard deviation	Median	Minimum	Maximum
Age	38.9	17.45	35	17	85
Days of stay	5.9	4.7	4	1	18
BMI	30.14	5.68	31	20	40
Glucose	113	43.8	103	61	242
Triglycerides	184	61.4	180	80	325
HDL cholesterol	43	13.08	40	20	80

**Table 5:** Mean age, days of hospital stay, BMI, glucose, triglycerides, and HDL cholesterol in patients with choledocholithiasis separated by presence or absence of MS MS: metabolic syndrome, BMI: body mass index

MS	Mean age	Mean days of hospital stay	Mean BMI	Mean glucose	Mean triglycerides	Mean HDL
No	32	6	24.3	90.8	125	56.3
Yes	42	5.85	32.8	124	211	36.9

Table 6 shows the frequency of MS in patients with choledocholithiasis broken down by variables; likewise, in each variable the p-value obtained through Fisher's exact test is demonstrated due to expected values less than 5 in the contingency tables to determine the statistically significant association between some of the variables and the presence of MS in patients with choledocholithiasis. Among the variables, a statistically significant relationship was found only between BMI and the presence of MS (p=0.0016). Specifically, the OR of BMI with MS was significant, indicating that having a high BMI (above 30) is a risk factor for developing MS in patients with choledocholithiasis.

**Table 6:** Frequency of MS and statistical tests broken down by variables in patients with choledocholithiasis BMI: body mass index, MS: metabolic syndrome, OR: odds ratio, CI: confidence interval * values are presented as N(%), ** A p-value of <0.05 was considered statistically significant

Variable	Presence of MS		P-value (Fisher's exact test)	OR (95% CI)	Association
	Yes*	No*			
Sex					
Female	8 (31%)	18 (69%)	0.8 (1.0)	0.89 (0.04-58.89)	No
Male	1 (33%)	2 (66%)			
Age (years)					
17-40	11 (62%)	7 (38%)	0.4118 (0.57)	2.76 (0.38-33.82)	No
Over 40	9 (82%)	2 (18%)			
BMI (kg/m^2^)					
18-30	5 (38%)	9 (64%)	0.0016** (11.14)	22 (2.18-1170.53)	Yes
30-40	14 (93%)	1 (7%)			

**Figure 1:**
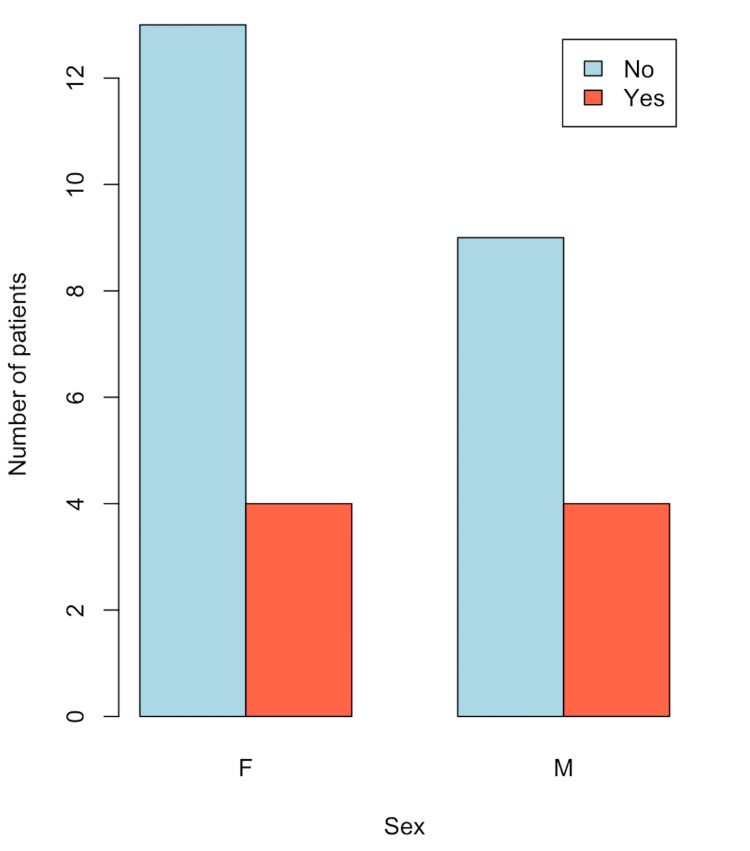
Frequency of MS by sex in patients with choledocholithiasis MS: metabolic syndrome

**Figure 2:**
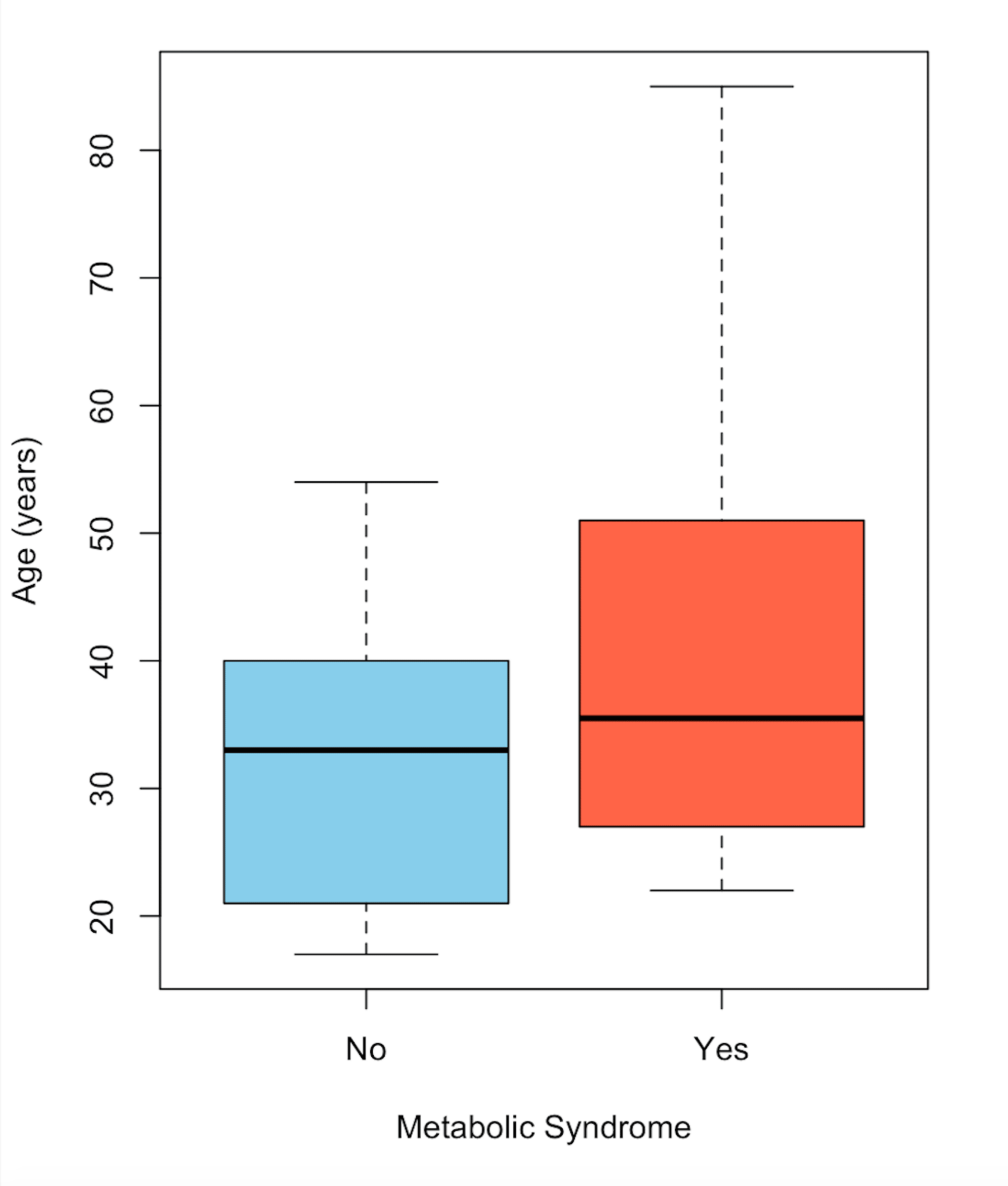
Frequency of MS by age in patients with choledocholithiasis MS: metabolic syndrome

**Figure 3:**
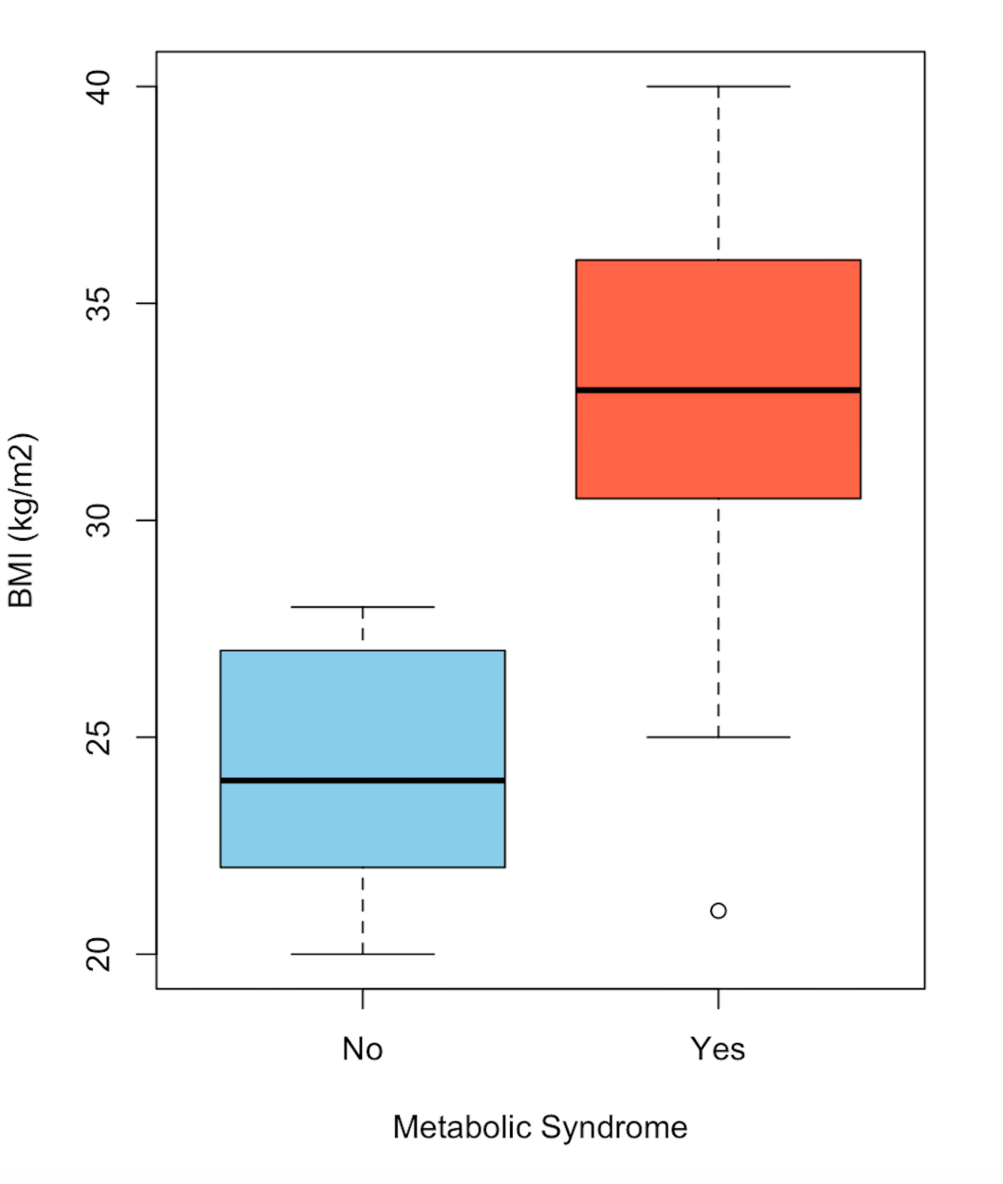
Frequency of MS by BMI in patients with choledocholithiasis MS: metabolic syndrome, BMI: body mass index

In Figure 1, the distribution of patients with choledocholithiasis, categorized by the presence or absence of MS and age, is observed. A higher proportion of women is noted compared to men. In Figure 2, a box plot of patients with choledocholithiasis, categorized by the presence or absence of MS, is shown by age, with the central line representing the mean of each group. The box represents 50% of the patients who are between the first and third quartiles (Q1-Q3), and the dotted lines represent the range of values; whereas, as shown, the means of both groups are similar; however, there is a variation between the quartiles and the data range. In Figure 3, a box plot of patients with choledocholithiasis, categorized by the presence or absence of MS according to BMI, is shown. A visual difference is evident in both the mean and the interquartile ranges (Q1 and Q3), as well as the range.

In Table 7, the descriptive analysis of age, days of hospital stay, BMI, glucose, triglycerides, and HDL cholesterol of patients without a diagnosis of choledocholithiasis is observed, in which a decrease in the means of all variables is observed, except for HDL cholesterol, which had an increase compared to Table 2 of patients with choledocholithiasis. In Table 8, the mean age, days of hospital stay, BMI, glucose, triglyceride levels, and HDL cholesterol levels are observed, divided by the presence or absence of MS in patients with a diagnosis of choledocholithiasis.

**Table 7:** Descriptive analysis of age, days of hospital stay, BMI, glucose, triglycerides, and HDL cholesterol in patients without choledocholithiasis BMI: body mass index, HDL: high-density lipoprotein

	Mean	SD	Median	Minimum	Maximum
Age	50.8	8.8	50	33	65
Days of hospital stay	4.17	1.15	4	2	6
BMI	28.6	5.01	29	20	40
Glucose	96.8	23.14	91.5	70	147
Triglycerides	140	44	126	90	245
HDL cholesterol	57	12.49	61	30	70

**Table 8:** Mean age, days of hospital stay, BMI, glucose, triglycerides, and HDL cholesterol in patients without choledocholithiasis separated by presence or absence of MS MS: metabolic syndrome, BMI: body mass index, HDL: high-density lipoprotein

MS	Mean age	Mean days of hospital stay	Mean BMI	Mean glucose	Mean triglycerides	Mean HDL
No	50.6	3.86	26.5	84.5	117	63.7
Yes	52.4	5	34.6	131	204	38.6

In Table 9, the frequency of MS in patients without choledocholithiasis is observed, broken down by variables; likewise, in each variable, the p-value obtained through Fisher's test is observed. Among the variables, only a statistically significant relationship was found between BMI and the presence of MS (p=0.008). For all variables, the odds ratio (OR) and its 95% CI were calculated. Only the OR for BMI with MS was significant, indicating that having a high BMI (above 30) is a risk factor for developing MS in patients without choledocholithiasis.

**Table 9:** Frequency of MS and statistical tests broken down by variables in patients without choledocholithiasis BMI: body mass index, MS: metabolic syndrome, OR: odds ratio, CI: confidence interval * values are presented as N(%), ** a p-value of <0.05 was considered statistically significant

Variable	Presence of MS		P-value (Fisher's exact test)**	OR (95% CI)	Association
	Yes*	No*			
Sex					
Female	13 (74%)	4 (26%)	0.977 (0.0007)	1.42 (0.20-9.97)	No
Male	9 (69%)	4 (31%)			
Age (years)					
17-40	1 (20)	4 (80%)	0.08612 (0.85)	1.84 (0.14-104)	No
Over 40	8 (32%)	17 (68%)			
BMI (kg/m^2^)					
18-30	2 (8%)	22 (92%)	0.008** (16.20)	41.6 (3.02-2644)	Yes
30-40	5 (83%)	1 (17%)			

**Figure 4:**
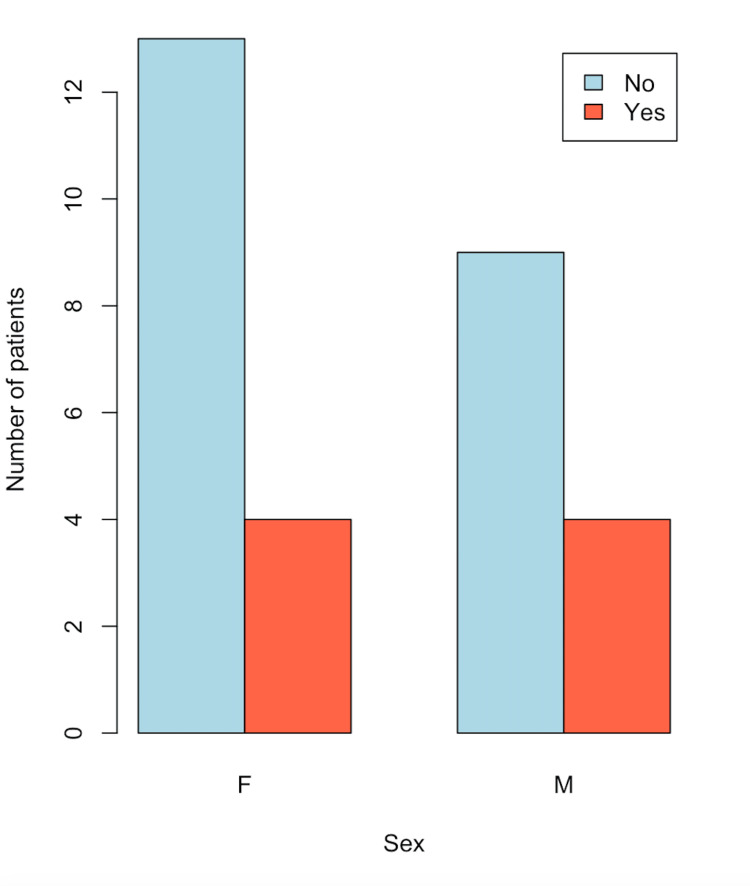
Frequency of MS by sex in patients without choledocholithiasis MS: metabolic syndrome

**Figure 5:**
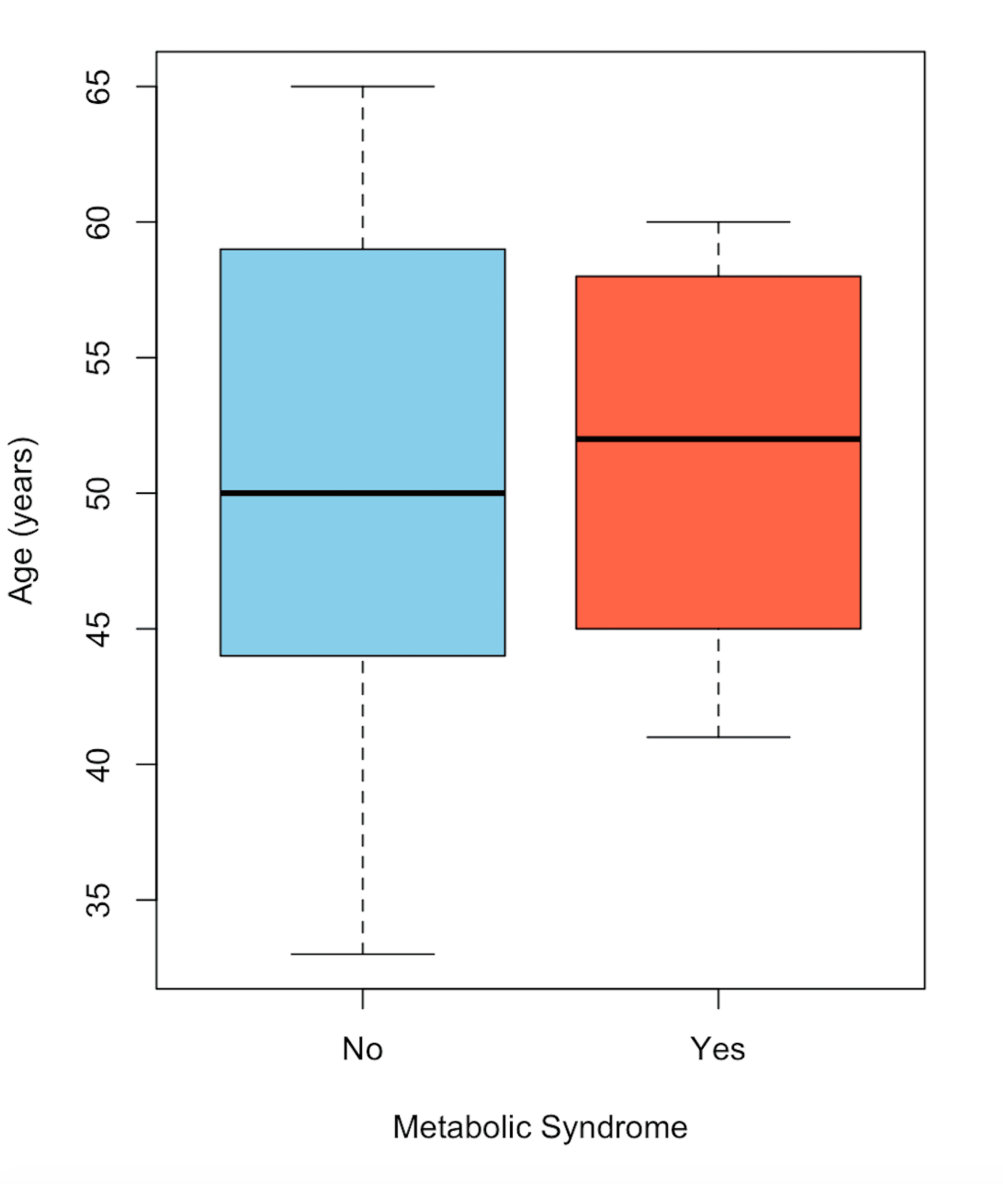
Frequency of MS by age in patients without choledocholithiasis MS: metabolic syndrome

**Figure 6:**
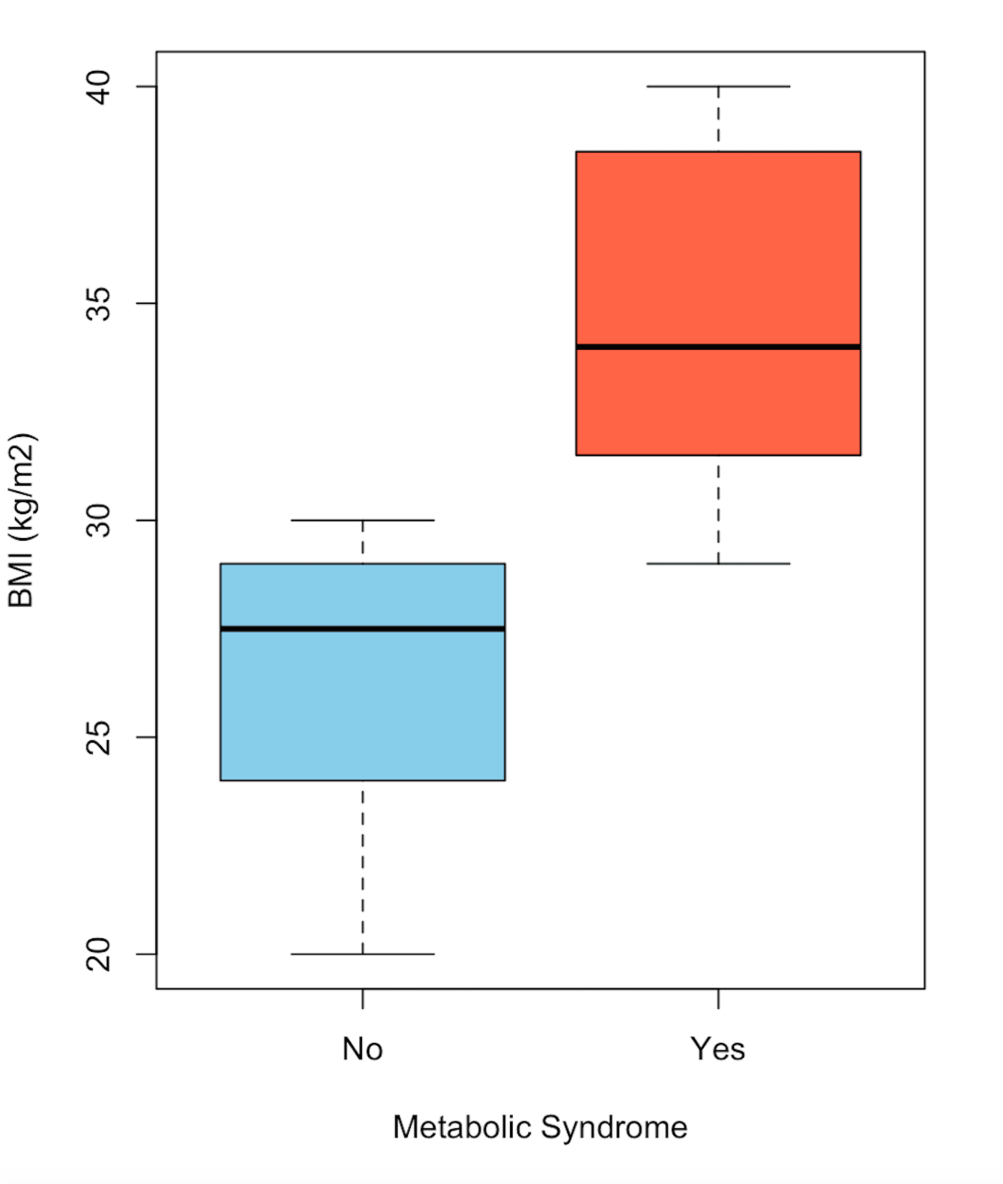
Frequency of MS by BMI in patients without choledocholithiasis MS: metabolic syndrome, BMI: body mass index

In Figure 4, the distribution of patients without choledocholithiasis, categorized by the presence or absence of MS and age, is observed. A similar proportion between men and women is noted, consistent with Figure 1. In Figure 5, a box plot of patients without choledocholithiasis, categorized by the presence or absence of MS, is shown by age. As seen, the means and interquartile ranges (Q1 and Q3) of both groups are similar, with a lower range in the group with MS. In Figure 6, a box plot of patients with choledocholithiasis, categorized by the presence or absence of MS according to BMI, is presented. A visual difference is observed in both the mean and the interquartile range (Q1 and Q3), as well as the total range.

## Discussion

The present research project demonstrates a statistically significant association between MS and choledocholithiasis, with an OR of 5.83 (95% CI: 1.93-19.33), which establishes that the presence of MS is a risk factor for the development of choledocholithiasis. There is limited evidence regarding the association of MS specifically with choledocholithiasis, as the available literature focuses on the presence of MS as a risk factor for biliary cholelithiasis (presence of stones in the gallbladder); however, despite this limitation, it is possible to extrapolate these results, since the individual components of MS, such as arterial hypertension, dyslipidemia, obesity, and diabetes, have been associated with a higher risk of cholelithiasis; and given that most patients with choledocholithiasis are secondary to the passage of stones previously formed in the gallbladder, results similar to ours can be expected when exclusively evaluating choledocholithiasis [6-8].

Our results showed that, in both patients with and without choledocholithiasis, BMI has a statistically significant association with the presence of MS (p=0.0016 and p=0.008, respectively), which is consistent with previous studies. The ATP III clinical criteria for the diagnosis of MS propose the use of abdominal circumference to determine central obesity; however, some studies assess the use of BMI greater than 30 kg/m² as a substitute for abdominal circumference, since this value is a better analogue to central obesity, especially compared to patients with BMI less than 30 kg/m², which is consistent with the results obtained both in patients with and without choledocholithiasis [9,10]. Obesity plays a significant role in the development of cholelithiasis and choledocholithiasis, as it promotes hepatic cholesterol synthesis, which increases the risk of developing certain biliary lithiasis diseases. In addition to also playing a role in the development of MS, we would have expected that in patients with choledocholithiasis there would be a higher OR compared to patients without choledocholithiasis; however, in the present work, a lower OR was found in patients without choledocholithiasis, which could be explained by having a low frequency at the time of making the contingency tables [11-13].

There is ambiguous literature about the role of age in the development of choledocholithiasis and MS. However, it is evidenced that the older the age, the greater the risk of suffering from metabolism-related diseases, such as MS. There is inconclusive evidence regarding age and its relationship with choledocholithiasis, since some mention that the older the age, the higher the prevalence of biliary lithiasis diseases, while in other studies no statistically significant relationship is found between age and the diagnostic probability of choledocholithiasis [14-17]. In our study, no statistically significant association was found between age and the presence of MS in either patients with or without choledocholithiasis, which was unexpected, as older age is associated with a greater probability of having a metabolic-related disease. The limited sample size could again explain this, or it may be because patients had undetected migratory stones or spontaneously eliminated them before diagnosis, which would create an underestimation of the actual prevalence of choledocholithiasis.

Among the limitations of this manuscript, the central one is that the small sample size (n=59) not only limits generalizability but also raises the possibility of type II error, where associations may have gone undetected due to insufficient statistical power; likewise, it is possible that the date range in which the sample was collected (January 2024-February 2025) limited the number of available cases, as well as their representativeness; additionally, the non-random, convenience sampling method used may introduce selection bias, potentially resulting in a sample that does not accurately reflect the broader patient population, and this could limit the external validity of the findings and may affect the ability to generalize the results to other settings, so these factors should be considered when interpreting the conclusions. In addition, the retrospective nature of the study entails intrinsic limitations, such as reliance on pre-existing medical records that may lack certain clinical details and the presence of unmeasured confounders that could influence the association being studied. Furthermore, it is possible that due to the migratory nature of gallstones, patients with choledocholithiasis were underdiagnosed, especially those with smaller or asymptomatic stones that may have resolved spontaneously without detection; however, despite these limitations, the present work represents a relevant contribution to the clinical field, being one of the few to explore the association between MS and choledocholithiasis specifically, where future studies could benefit from having a prospective design, a broader data collection period, and longitudinal follow-up. Expanding research to multicenter cohorts, integrating imaging follow-up protocols, and incorporating biochemical or genetic markers of metabolic dysfunction may also strengthen the evidence base and refine patient stratification, so the continuation of this line of research would allow the establishment of more specific prevention strategies, as well as a more comprehensive clinical approach in patients with metabolic risk.

## Conclusions

The present study achieved its objectives by evaluating the potential association between MS and choledocholithiasis, finding a statistically significant relationship between the two conditions. Based on the results, the null hypothesis was rejected, and it was established that MS is a relevant risk factor for the development of choledocholithiasis. However, these findings should be interpreted with caution due to the study’s limitations, including a small sample size and retrospective design. Nonetheless, the results highlight the potential importance of reinforcing preventive strategies and considering early screening for biliary pathology in patients with MS, particularly those presenting with multiple risk factors.
